# Data set of differentially expressed microRNAs in sanguinarine-treated *Caenorhabditis elegans* and its F3 progeny

**DOI:** 10.1016/j.dib.2018.10.047

**Published:** 2018-10-23

**Authors:** Yit-Lai Chow, Fumihiko Sato

**Affiliations:** Division of Integrated Life Science, Graduate School of Biostudies, Kyoto University, Kitashirakawa, Sakyo, Kyoto 606-8502, Japan

**Keywords:** *Caenorhabditis elegans*, Sanguinarine, MicroRNA, Benzylisoquinoline alkaloids, Transgenerational inheritance

## Abstract

This article presents small RNA sequencing data of *Caenorhabditis elegans* consist of P0 control worms (untreated), P0 worms treated with a plant alkaloid, sanguinarine, and its F3 offspring. The data were analyzed to identify microRNAs that were differentially expressed in both the sanguinarine-treated P0 and its descendants F3 worms. Targets of the identified miRNAs, gene function annotations and their functional clusters are shown. The data presented here will facilitate comparison with data from other researchers who are working on miRNAs profiling of xenobiotic-treated *C. elegans*.

**Specifications table**

**Table:** 

Subject area	*Molecular biology*
More specific subject area	*miRNAs* in *Caenorhabditis elegans*
Type of data	*Table, figure*
How data was acquired	*Small RNA sequencing using Illumina HiSeq. 2000.*
Data format	*Analyzed data*
Experimental factors	*Comparison of wild-type N2 C. elegans P0 control against P0 worms treated with sanguinarine and their F3 offspring.*
Experimental features	*Wild-type N2 P0 worms were treated with or without 10 μM sanguinarine for 48 h. RNA from those worms and F3 of treated P0 worms were extracted for small RNA sequencing.*
Data source location	*Kyoto University, Kitashirakawa, Sakyo, Kyoto, Japan*
Data accessibility	*Data is provided with this article.*
	*Small RNA sequencing data were deposited in DNA Data Bank of Japan (DDBJ) with the accession number DRA006564.*
	http://ddbj.nig.ac.jp/DRASearch/submission?acc=DRA006564

**Value of the data**

• Transgenerational effects of xenobiotics have not been investigated much despite its importance in understanding the epigenetic inheritance of drug׳s effect on the descendants of people who took the drugs as medicine. Small RNA transcriptome analysis could help identify the heritable small RNAs and their targeted genes that can affect the genes expressions and physiology of the future generations.
• The data set generated for control, sanguinarine-treated and its F3 generation offspring of the *Caenorhabditis elegans* helps in identifying differentially expressed genes and related cellular pathways, and provides an insight to the epigenetic inheritance of miRNAs in the offspring of alkaloid-treated worms.
• The data set can be used by researchers for comparison to other studies about xenobiotic effects on miRNA expressions.

## 1 Data

**Fig. 1:**
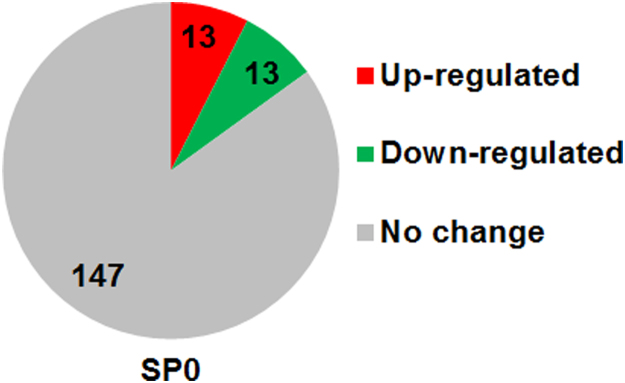
Number of annotated miRNAs found in sanguinarine-treated P0 worms. Up-regulated and down-regulated miRNAs refers to differential expression for ≥2 fold-change compared to P0 control.

**Fig. 2:**
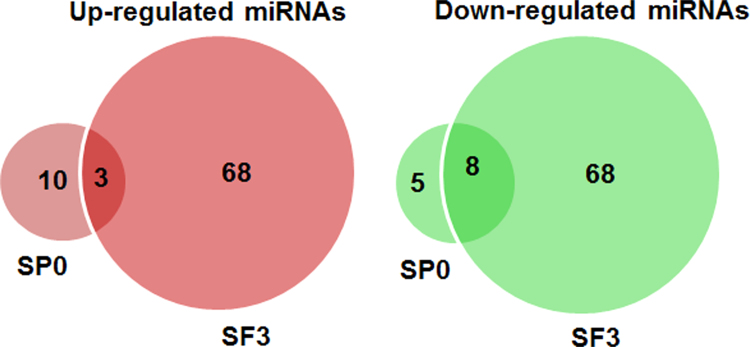
Number of annotated miRNAs found in sanguinarine-treated P0 (SP0) and its descendants F3 (SF3) worms. Up-regulated and down-regulated miRNAs refers to differential expression for ≥2 fold-change compared to control.

**Fig. 3:**
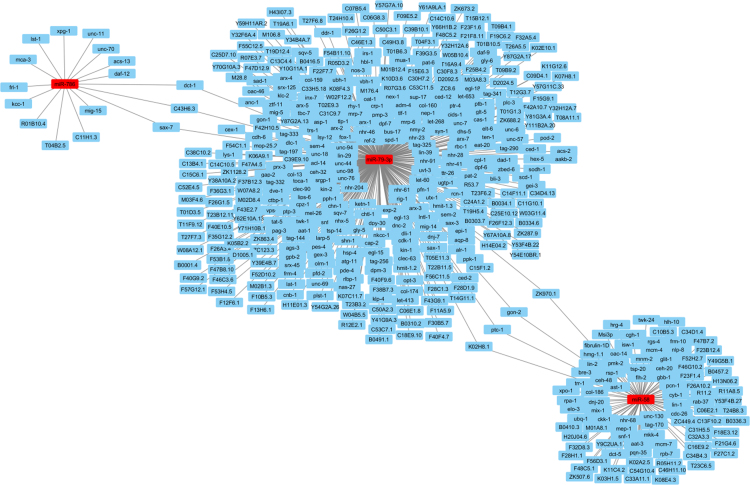
Up-regulated miRNAs in both SP0 and SF3 with their target genes.

**Fig. 4:**
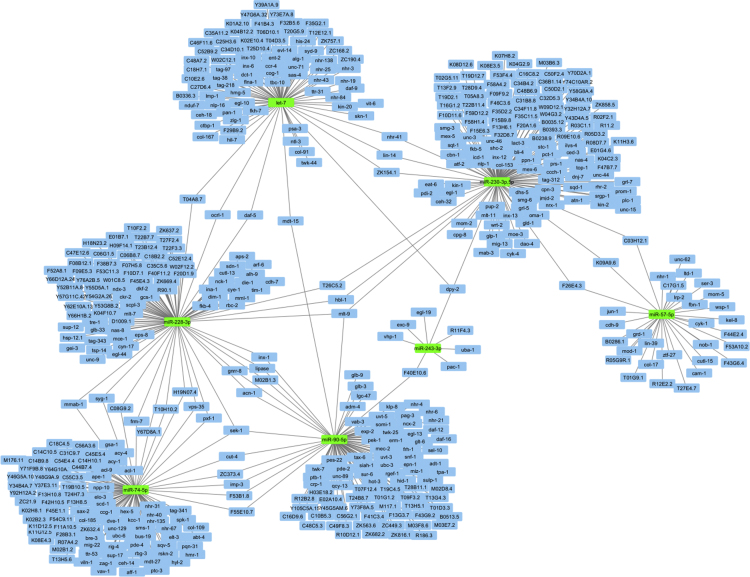
Down-regulated miRNAs in both SP0 and SF3 with their target genes.

This article contains analysis from small RNA sequencing data of *Caenorhabditis elegans* consist of P0 control worms (untreated), P0 worms treated with a plant alkaloid, sanguinarine, and its F3 offspring. Fig. 1 shows the number of identified miRNAs in sanguinarine-treated P0 (SP0) whereby the number of up-regulated and down-regulated miRNAs indicates ≥2 fold-change differential expression compared with control P0 worms. Sequences that correspond to the predicted mature miRNAs were listed in Tables 1 and 2. The data were further analyzed to identify microRNAs that were differentially expressed in both the sanguinarine-treated P0 and its descendants F3 worms (Fig. 2). The interaction of differentially expressed miRNAs with their target genes were plotted using Cytoscape open source software ( Figs. 3 and 4). Data of differentially expressed miRNAs were analyzed using DAVID analysis software to obtain the miRNAs target gene function annotations (for biological processes), enrichment scores and functional clusters ( Tables 3 and 4). Details of gene ontology, functional annotations, clusters and fold enrichment are attached as Supplementary files 1 and 2.

**Table 1:** Up-regulated miRNAs with more than 2 fold change in P0 sanguinarine-treated *C. elegans* (SP0) compared to P0 control.

SP0 up-regulated	Fold change (+)
Cel-miR-234-3p	2.001
Cel-miR-79-3p	2.024
Cel-miR-392-3p	2.041
Cel-miR-1-3p	2.058
Cel-miR-83-5p	2.060
Cel-miR-254-5p	2.097
Cel-miR-58a-3p	2.101
Cel-miR-2-3p	2.102
Cel-miR-796	2.172
Cel-miR-67-3p	2.236
Cel-miR-786-5p	2.257
Cel-miR-792-3p	2.307
Cel-miR-2220-3p	2.511

**Table 2:** Down-regulated miRNAs with more than 2 fold change in P0 sanguinarine-treated *C. elegans* (SP0) compared to P0 control.

SP0 down-regulated	Fold change (-)
Cel-miR-789-2-5p	−2.082
Cel-miR-243-3p	−2.089
Cel-miR-41-5p	−2.099
Cel-miR-57-5p	−2.115
Cel-miR-80-5p	−2.125
Cel-miR-35-3p	−2.130
Cel-miR-74-5p	−2.138
Cel-miR-230-5p	−2.139
Cel-miR-90-5p	−2.628
Cel-miR-228-3p	−2.856
Cel-miR-230-3p	−3.092
Cel-miR-37-5p	−3.198
Cel-let-7-3p	−9.222

**Table 3:** Functional annotation clustering of up-regulated genes by DAVID system.

Annotation cluster	Enrichment score	Functional annotation
1	3.983917003376599	• Arp2/3 complex-mediated actin nucleation • epithelial cell migration • morphogenesis of embryonic epithelium • regulation of actin filament polymerization • endocytosis
2	2.8016223249876955	• Zinc finger, C2H2, C2H2-like • Zinc finger C2H2-type/integrase DNA-binding domain
3	2.0850944843257153	• Glycosyl transferase, family 2 • protein glycosylation • Cell envelope biogenesis, outer membrane
4	1.951189021926856	• Immunoglobulin I-set, Immunoglobulin subtype 2 • Immunoglobulin-like domain, fold • Fibronectin, type III
5	1.8941659421354167	• Tetratricopeptide repeat-containing domain • Tetratricopeptide-like helical
6	1.36177557664346	• Amino acid transmembrane transport • Amino acid/polyamine transporter I • L-amino acid transport • L-alpha-amino acid transmembrane transport
7	1.1354750862981595	• Major facilitator superfamily, domain • Anion transport
8	1.1069070652173487	• mitotic nuclear division • cell division • cell cycle
9	0.9483517008551856	• DNA replication • Nucleic acid-binding, OB-fold
10	0.8716391502323106	• Homeodomain, Homeodomain-like • Homeobox, conserved site
11	0.5851361303274127	• Small GTPase superfamily • Small GTP-binding protein domain • small GTPase mediated signal transduction
12	0.429270506932433	• Zinc finger, NHR/GATA-type • Steroid hormone receptor • Zinc finger, nuclear hormone receptor-type • Nuclear hormone receptor, ligand-binding, core • regulation of transcription, DNA-templated • transcription, DNA-templated steroid hormone mediated signaling pathway
13	0.41869272211420305	• Protein kinase, ATP binding site • Serine/threonine-protein kinase, active site • Protein kinase, protein kinase-like domain, catalytic domain • protein phosphorylation
14	0.3419388621013877	• WD40/YVTN repeat-like-containing domain • WD40-repeat-containing domain
15	0.29648404116073784	• RNA recognition motif domain • Nucleotide-binding, alpha-beta plait • mRNA processing
16	0.008889837572459433	• G protein-coupled receptor, rhodopsin-like • GPCR, rhodopsin-like, 7TM • G-protein coupled receptor signaling pathway

**Table 4:** Functional annotation clustering of down-regulated genes by DAVID system.

Annotation cluster	Enrichment score	Functional annotation
1	3.2517084196213317	• regulation of transcription, DNA-templated • Zinc finger, NHR/GATA-type • Zinc finger, nuclear hormone receptor-type • Nuclear hormone receptor, ligand-binding, core steroid hormone mediated signaling pathway
2	2.633095835439009	• Homeodomain, Homeodomain-like • Homeobox, conserved site
3	2.18048137148177	• Peptidase M12B, ADAM/reprolysin, propeptide • Blood coagulation inhibitor, Disintegrin • Metallopeptidase, catalytic domain • proteolysis
4	1.8316625707281753	• Immunoglobulin subtype, 2 • Immunoglobulin-like fold, Immunoglobulin-like domain • Immunoglobulin I-set
5	1.4131446761832307	• Guanine-nucleotide dissociation stimulator CDC25 • Ras guanine nucleotide exchange factor, domain • small GTPase mediated signal transduction
6	1.3595435387017165	• Cadherin conserved site • Cadherin-like homophilic cell adhesion via plasma membrane adhesion molecules
7	1.2567261547011277	• interneuron migration, motor neuron migration, neuroblast migration, neuron migration • Wnt signaling pathway
8	1.1634084614953117	• Insulin-like growth factor binding protein, N-terminal • EGF-type aspartate/asparagine hydroxylation site • Epidermal growth factor-like domain • EGF-like; EGF-like calcium-binding, conserved site
9	1.0723210327161894	• Protein kinase, ATP binding site • Serine/threonine-protein kinase, active site • Protein kinase, catalytic domain, Protein kinase-like domain • protein phosphorylation
10	1.002675959580202	• EF-hand-like domain, EF-hand domain • EF-Hand 1, calcium-binding site
11	0.8140390002380976	• Mitochondrial substrate/solute carrier, carrier domain • Translation
12	0.6518268930200478	• Zinc finger C2H2-type/integrase DNA-binding domain • Zinc finger, C2H2-like Zinc finger, C2H2
13	0.5872305866210232	• Thioredoxin, conserved site • protein folding • Thioredoxin domain, Thioredoxin-like fold • cell redox homeostasis
14	0.5300622856853572	• potassium ion transmembrane transport • Two pore domain potassium channel • Ion transport 2 • stabilization of membrane potential
15	0.2075133112483454	• Protein-tyrosine phosphatase, active site • Protein-tyrosine/Dual specificity phosphatase • protein dephosphorylation • peptidyl-tyrosine dephosphorylation
16	0.05598435901395593	• WD40 repeat • WD40-repeat-containing domain • WD40/YVTN repeat-like-containing domain
17	0.04588463542842712	• G protein-coupled receptor, rhodopsin-like • GPCR, rhodopsin-like, 7TM • G-protein coupled receptor signaling pathway

Three miRNAs up-regulated in SP0 and SF3 worms were: miR-79-3p; miR-58a-3p; miR-786-5p. Eight miRNAs down-regulated in SP0 and SF3 worms were: let7-3p; miR-243-3p: miR-57-5p; miR-74-5p; miR230-3p; miR-230-5p; miR-90-5p; miR-228-3p.

## 2 Experimental design, materials and methods

### 2.1 Worm culture

Wild-type N2 (Bristol) worms were maintained on nematode growth media (NGM) at 20 °C according to standard culture methods [1].

### 2.2 Worm treatment

L4 larval stage worms were treated with 10 μM sanguinarine or distilled water (as control) for 48 h at 20 °C, 180 rpm (for aeration) in liquid S-Medium with *Escherichia coli* OP50 as food source in 24-well plates. After 48 h, control worms and sanguinarine-treated worms were collected, rinsed in M9 buffer and pelleted for RNA extraction as previously reported [2]. 20 worms from the sanguinarine-treated group were randomly picked and transferred to fresh NGM plates with OP50 and allowed to lay eggs. The procedure was repeated until F3 worms reached adult stage (at the same growth stage as P0 worms) and were collected for RNA extraction.

### 2.3 RNA samples

Total RNA was extracted with Sepasol-RNA I Super G (Nacalai Tesque), purified with an RNeasy Mini Kit (Qiagen). The concentration and purity of the RNA was determined using NanoDrop Spectrophotometer (Thermo Scientific 2000). The integrity of the extracted RNA was analyzed on a Bioanalyzer (Agilent Technologies 2100). P0 Control, sanguinarine-treated P0 and its F3 offspring RNA samples with RNA integrity numbers: 8.6, 10, 10 respectively, were used for library preparation. Sequencing was carried out using Illumina HiSeq. 2000 platform, single-end, 50 basepair read length.

### 2.4 Differentially expressed miRNAs

The differentially expressed miRNAs between P0 control and P0 sanguinarine-treated were determined using log_2_ fold change and results with ≥2.0 fold change versus control were used for further analysis. The data were aligned to miRBase database (www.mirbase.org) and target predictions were obtained from TargetScanWorm database (www.targetscan.org). Interaction of the miRNAs with their target genes were plotted using Cytoscape v. 3.4.0 software [3].

### 2.5 Functional annotation clustering and enrichment scores

Only the miRNAs that are both up-regulated or both down-regulated in P0 sanguinarine-treated and F3 of sanguinarine-treated P0 versus P0 control were analyzed. Functional annotation clustering (medium stringency) and gene ontology (biological processes) enrichment scores were processed using DAVID v. 6.8 system [4].
